# Combined Ingestion of Tea Catechin and Citrus β-Cryptoxanthin Improves Liver Function via Adipokines in Chronic Obesity

**DOI:** 10.3390/nu15153345

**Published:** 2023-07-27

**Authors:** Kazuhiko Nakadate, Kiyoharu Kawakami, Noriko Yamazaki

**Affiliations:** 1 Department of Basic Science, Educational and Research Center for Pharmacy, Meiji Pharmaceutical University, 2-522-1, Noshio, Kiyose 204-8588, Tokyo, Japan; d236953@std.my-pharm.ac.jp; 2 Department of Community Health Care and Sciences, Meiji Pharmaceutical University, 2-522-1, Noshio, Kiyose 204-8588, Tokyo, Japan; nyamazak@my-pharm.ac.jp

**Keywords:** adipokines, activin E, green tea, catechin, β-cryptoxanthin, polyphenol, flavonoid, β-carotene, obesity, high-calorie

## Abstract

Recently, there has been an increase in the number of obese individuals, which has elevated the risk of related diseases. Although several studies have been performed to develop a definitive treatment for obesity, no solution has yet been achieved. Recent evidence suggests that tea catechins possess antiobesity effects; however, an impractical amount of catechin may be required to achieve antiobesity effects in humans. Moreover, studies are yet to elucidate the effects of the combined treatment of tea catechins with other substances. Here, we investigated the synergistic effects of catechins and β-cryptoxanthin in high-calorie diet-induced mice. Combined treatment with catechins and β-cryptoxanthin significantly suppressed obesity-induced weight gain and adipocyte size and area, restoring serum parameters to normal. Additionally, combined treatment with catechins and β-cryptoxanthin suppressed inflammatory responses in adipocytes, restored adiponectin levels to normal, protected the liver against obesity-induced damage, and restored normal liver function. Moreover, activin E level was restored to normal, possibly affecting the energy metabolism of brown adipocytes. Overall, these results suggest that the combined ingestion of tea catechins and β-cryptoxanthin was not only effective against obesity but may also help to prevent obesity-related diseases, such as diabetes and cardiovascular diseases.

## 1. Introduction

In recent decades, there has been an annual increase in the number of people with chronic obesity in several countries [1,2], elevating the risk of associated diseases. The current treatment for obesity includes exercise therapy and dietary modifications in combination with medications; however, these treatments have only been partially effective. In addition to clinical studies, animal models have been used for basic research on chronic obesity [3,4,5]. For instance, the administration of monosodium glutamate (MSG) immediately after birth can be used to develop animal models of chronic obesity [6,7,8]. Since the hypothalamus is still immature immediately after birth, administered MSG crosses the blood–brain barrier (BBB) and flows into the hypothalamus, destroying nerve cells associated with satiety and the bioenergetic control center [9,10]. Several studies have been conducted using the MSG-administered animal model, especially to examine the effects of chronic obesity on liver and brain function [11,12,13]. Chronic obesity is linked to an increase in adipose tissue, which produces and secretes various adipokines such as adiponectin, leptin, and tumor necrosis factor-alpha (TNF-α) while also storing energy as neutral fat [14,15,16]. Macrophages located in adipose tissue are implicated in inflammation and can be divided into two categories: M1 and M2 macrophages [17,18,19], each exhibiting different properties in adipose tissues [20]. The number of M1 macrophages increases with obesity, and these cells secrete pro-inflammatory cytokines that lead to inflammatory changes in adipose tissue. In contrast, M2 macrophages reduce inflammatory changes in nonobese adipose tissues by secreting anti-inflammatory cytokines. Additionally, adipokines, particularly adiponectin, can affect liver metabolism via the adiponectin receptor. Adiponectin can increase the expression of ACO and mitochondrial uncoupling protein (UCP), which are involved in energy consumption [21]. Moreover, there is a decrease in the expression of adiponectin receptor type 1 (AdipoR1) and adiponectin receptor type 2 (AdipoR2) in animal models of obesity and type 2 diabetes, which is one of the causes of diabetes [22]. A previous study showed that activin E secreted from the liver affects brown adipocytes and enhances heat production [23]. Additionally, liver activin E overexpression activated thermogenesis through UCP upregulation in brown and white adipose tissue and improved insulin sensitivity in liver activin E transgenic mice [23]. Conversely, inhibin βE gene silencing inhibited thermogenesis in adipose tissue [23]. Based on these findings, it could be speculated that adiponectin secreted from white adipose tissue affects hepatocytes by promoting activin E secretion, which increases energy consumption in brown adipocytes, leading to an increase in body fat and improvement in glucose metabolism.

Recently, studies have been conducted to assess the efficacy of several experimental drugs in the treatment of chronic obesity [24,25]. For instance, tea catechins, which possess antioxidant effects, have been shown to suppress chronic obesity [26,27]. Additionally, long-term intake of tea catechins was effective in suppressing weight gain and improving chronic obesity [28]. However, to verify the obesity-reducing effects of tea catechins in humans, ingesting a large amount or high concentration of tea catechins is required, which is unrealistic. Therefore, we recently investigated the synergistic effects of foods that exert the beneficial effects of tea catechins using an MSG mouse model. Specifically, a combination of β-cryptoxanthin and low concentrations of tea catechins exerted a significant obesity-reducing effect [29], decreased M1 macrophage levels, and restored M2 macrophage levels to normal.

Although the MSG mouse model is extremely useful for examining pathological conditions, it is unsuitable for extrapolating chronic obesity associated with a high-calorie diet in humans. Here, we examined the antiobesity effects and mechanism of tea catechins and β-cryptoxanthin in a mouse model of chronic obesity generated using a high-fat diet. Specifically, we investigated the possibility that combined treatment with tea catechins and β-cryptoxanthin may increase adipokine secretion from adipose tissue and improve the pathological conditions of hepatocytes, further enhancing activin E secretion from hepatocytes.

## 2. Materials and Methods

### 2.1. Animals

Male C57BL/6J mice were utilized in this study. Six mother mice with eight male mice (post-natal day 0) were acquired from Japan SLC Co., Ltd. in Tokyo, Japan. The animals were kept with their mothers in a single cage until weaning at 3 weeks old. After weaning, they were moved to individual cages, with two mice per cage. The mice were kept in a controlled environment (temperature, 23 ± 2 °C; humidity, 55 ± 10%; 12 h light–dark cycle) and had unlimited access to food and water.

The Laboratory Animal Ethics Committee of the Meiji Pharmaceutical University granted approval for this study (No. 2707, 1 April 2020–2022). All animal handling and experimental protocols were conducted in accordance with Meiji Pharmaceutical University’s guidelines for animal experimentation.

### 2.2. Generation of Obese Mice

A mouse model of obesity was generated following previously described procedures [11,29,30]. Eight male mice were subcutaneously injected with MSG (Wako Pure Chemical Industries Ltd., Tokyo, Japan) at 1, 2, 4, 6, 8, and 10 d of age (MSG group) at a dosage of 2 mg/kg body weight. To generate a high-fat diet-induced chronic obesity model, 32 mice were fed a high-fat diet (D12492 ultra-high-fat feed with 60% kcal fat content, EPS EKISHIN Co., Ltd., Tokyo, Japan) after weaning on post-natal day 21. Eight mice were maintained on a control diet (D12450B Research Diets for Control, EPS EKISHIN Co., Ltd., Tokyo, Japan; control group).

### 2.3. Weight Measurement and Measurement of Food and Water Intake

In accordance with prior studies [12,29,30], all mice were weighed at a fixed time (noon) each week after 3 weeks of weaning. Additionally, the amount of food consumed in each cage was monitored daily during weeks 11 to 15 at a fixed time (noon), and the average daily amount of food per animal was calculated [11,12]. Similarly, the amount of water consumed in each cage was monitored daily during weeks 11 to 15 at a set time (noon), and the average daily water intake per animal was calculated.

### 2.4. Administration of Green Tea Catechins and/or Β-Cryptoxanthin

In accordance with previous studies [29,31,32], green tea catechins and/or β-cryptoxanthin were orally administered for 4 weeks, from 11 to 15 weeks of age. Green tea catechin (Sigma-Aldrich Japan, Tokyo, Japan) was diluted in water and administered daily at a dose of 1.7 mg/kg, which is equivalent to half the concentration effective in humans (200 mg/60 kg) [33,34]. The green tea catechin used in this experiment contains the following ingredients: caffeine, (+)-catechin, (−)-catechin 3-gallate, (−)-epicatechin, (−)-epicatechin-3-gallate, (−)-epigallocatechin 3-gallate, (−)-gallocatechin, and (−)-gallocatechin 3-gallate. Similarly, β-cryptoxanthin (Wako Pure Chemical Industries Ltd., Tokyo, Japan) was dissolved in water and administered daily at a dose of 50 mg/kg body weight, as previously reported [29,35].

### 2.5. Blood Biochemistry Analysis

Blood samples were collected from all the mice at 15 weeks of age and placed into 1.5 mL tubes, which were then mixed by inverting them. Subsequently, the tubes were kept at room temperature (RT) for 30 min to allow blood clotting. They were then centrifuged at 1500× *g* for 20 min to obtain the serum, which was promptly frozen. Glucose, total protein, total lipid, low-density lipoprotein (LDL) cholesterol, high-density lipoprotein (HDL) cholesterol, free cholesterol, non-esterified fatty acid (NEFA), triglyceride (TG), alkaline phosphatase (ALP), aspartate aminotransferase (AST), and alanine aminotransferase (ALT) levels were determined using appropriate test kits (Wako Pure Chemical Industries Ltd., Osaka, Japan).

### 2.6. Enzyme-Linked Immunosorbent Assay (ELISA)

After collecting the blood, the mice were anesthetized, and internal fat tissues were obtained. The adipose tissues from each mouse were quickly homogenized in ice-cold cell extraction buffer (Abcam, Cambridge, UK). The homogenates were left to incubate on ice for 20 min, centrifuged at 18,000× *g* for 20 min at 4 °C, and carefully rinsed with phosphate-buffered saline (PBS) to remove any blood. The supernatants were moved to fresh tubes, and the pellets were discarded. Samples were either used right away in the tests or divided into aliquots and stored at −80 °C.

Samples were analyzed using the following ELISA kits: mouse Arginase 1 ELISA Kit, mouse TNF alpha ELISA Kit, mouse IL-6 ELISA Kit, mouse IL-10 ELISA Kit, mouse IL-1 beta ELISA Kit, and mouse adiponectin ELISA Kit, all obtained from Abcam (Cambridge, UK). Briefly, 50 μL of each sample was added to the respective wells, followed by the addition of 50 μL of each antibody and thorough mixing. The plates were then sealed and placed on a plate shaker at 400 rpm, incubating for 1 h at RT. Subsequently, each well was washed three times with 350 μL of 1× wash buffer PT. Next, 100 μL of TMB development solution was added to each well, and the plate was incubated in the dark on a plate shaker at 400 rpm for 10 min. Finally, 100 μL of the Stop Solution was added to each well and mixed on a plate shaker for 1 min. The optical density was measured at 595 nm using a microplate reader (Infinite F50 plus, Tecan, Zürich, Switzerland).

### 2.7. Histological Analysis

After blood sampling, the mice were anesthetized, and internal adipose and liver tissues were collected. The adipose and liver tissues were immersed and fixed in a 4% paraformaldehyde solution (pH 7.4) for 2 days. Subsequently, the tissues were cut in half, washed with phosphate buffer, and subjected to a dehydration series (50%, 70%, 80%, 90%, 95%, and 100% ethanol), followed by immersion in Lemosol A (Wako Pure Chemical Industries Ltd., Tokyo, Japan). The tissues were then embedded in paraffin. The paraffin-embedded tissues were sliced into 5 μm thick sections using a sliding microtome (REM-710, Yamato Kohki Industrial, Tokyo, Japan), deparaffinized, and stained with hematoxylin–eosin (HE) solution (Muto Pure Chemicals Co., Ltd., Tokyo, Japan). After washing, the sections were dehydrated with increasing concentrations of ethanol and Lemosol A (Wako Pure Chemical Industries Ltd., Tokyo, Japan), and a cover slip was applied.

For immunostaining, the paraffin-embedded liver tissues were cut into 5 μm thick sections using a sliding microtome. Subsequently, the sections were mounted on slide glass, deparaffinized, and dehydrated in graded concentrations of ethanol. After rinsing with distilled water, antigen inactivation treatment was performed using citrate buffer (pH 6.0) at 95 °C for 90 min. After rinsing with PBS containing 0.3% Triton X-100 (PBS-T), the sections were incubated with blocking solution (normal goat serum, Vector Laboratories, CA, USA) in PBS-T at RT for 45 min, followed by incubation with primary antibodies against adiponectin receptor (14361-1-AP, diluted 1:500, Proteintech Japan, Tokyo, Japan) or INHBE (component of Actin E, NBP1-89262, diluted 1:500, Novus Biologicals, CO, USA) overnight at 4 °C. After washing with PBS, the sections were incubated with Alexa-488-conjugated anti-rabbit antibody (diluted 1:500, Abcam, Cambridge, UK) at RT for 45 min, washed, and cover-slipped.

For Oil-Red O staining, the half-sectioned portion of the middle lobe of the liver was cut into 1 mm thick slices, immersed in a 30% sucrose solution, and rapidly frozen with liquefied carbon dioxide gas. Subsequently, the frozen tissue sections were cut into 30 μm thick sections using a sliding microtome and stained with an Oil-Red O staining solution. The sections were mounted on slide glass and cover-slipped.

The tissue sections were viewed with an optical microscope (BZ-X700, Keyence, Osaka, Japan), and pictures were taken.

### 2.8. Statistical Analysis

The size of white adipocytes was measured using Image J software (version 1.54). We analyzed five photographs from one mouse in each group and measured a total of 150 adipocytes in each group. The data from each experiment are presented as the mean ± standard deviation (SD). Statistical differences were determined using analysis of variance (ANOVA), and a significance level of *p* < 0.05 was established. All statistical analyses were conducted using Microsoft Excel and StatView statistical software (version 5.0.1, SAS Institute Inc., Cary, NC, USA).

## 3. Results

### 3.1. Body Weight and Food and Water Intake at 15 Weeks of Age

**Figure 1:**
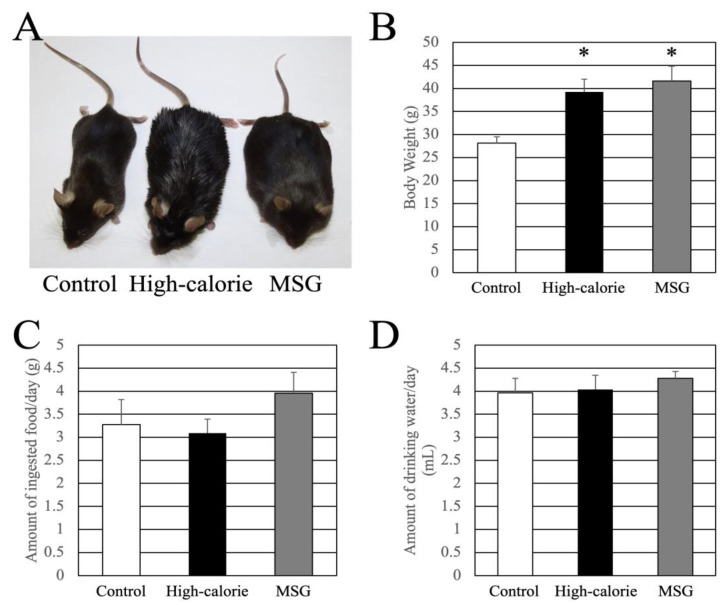
(**A**) Photo of mice (15 weeks of age) in each group. (**B**) Average body weight (15 weeks of age) of mice in each group. Average daily (**C**) feed intake and (**D**) water intake (15 weeks of age) in each group. Data are presented as mean ± standard deviation (SD); * *p* < 0.05 compared to control animals.

Mice models of chronic obesity were generated through MSG administration and by providing a high-fat diet [11,30]. The weight gain, feed consumption, and water intake of the mice at 15 weeks of age are depicted in Figure 1. Compared to the control group, mice in the MSG and high-calorie diet groups exhibited significantly greater weight gain (Figure 1A,B), indicating obesity. However, there were no significant differences in the daily feed and water intake among the groups (Figure 1C,D).

### 3.2. Changes in Body Weight and Blood Biochemistry

**Figure 2:**
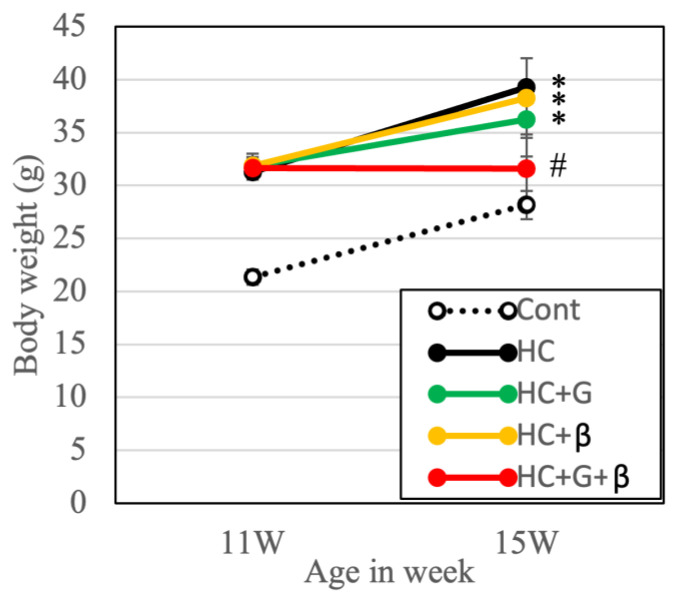
Changes in the body weight (11–15 weeks of age) of mice in each group. Cont, HC, HC + G, HC + β, and HC + G + β indicate the control, high-calorie-fed, high-calorie-fed + green-tea-intake, high-calorie-fed + β-cryptoxanthin-intake, and high-calorie-fed + green tea + β-cryptoxanthin-intake groups, respectively. Data are presented as mean ± standard deviation; * *p* < 0.05 compared to the control group, and ^#^ *p* < 0.05 compared to high-calorie-fed group.

To examine the effect of obesity on blood chemistry and body weight, high-calorie-fed obese mice (HC) that reached 11 weeks of age were divided into four groups (Figure 2): the water administration group (control), the tea catechin group (HC + G), the β-cryptoxanthin group (HC + β), and the tea catechin and β-cryptoxanthin group (HC + G + β). Throughout the experimental period (weeks 11–15), mice in the HC, HC + G, and HC + β groups exhibited significantly higher weight gain compared to the control group, with a steady increase in body weight observed in all groups except the HC + G + β group, which remained stable. At the end of the experiment, mice in the HC, HC + G, and HC + β groups had significantly higher body weights compared to the HC + G + β group. Importantly, there was no significant difference in body weight between mice in the HC + G + β and control groups after the 4-week treatment period, indicating that combined treatment with β-cryptoxanthin and catechin inhibited obesity-induced weight gain (Figure 2).

**Figure 3:**
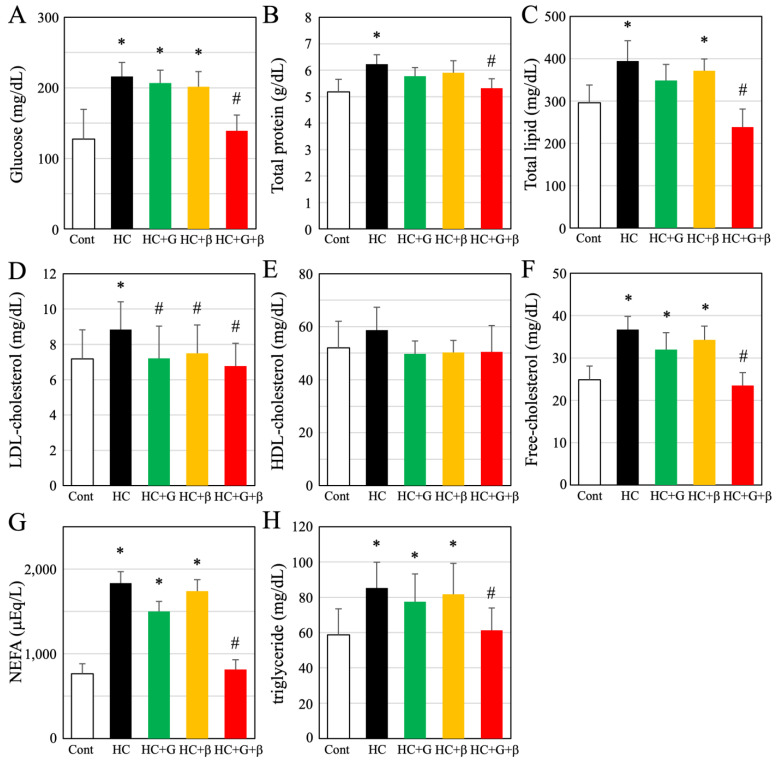
Blood chemistry of mice in the different groups. (**A**) Blood glucose, (**B**) total protein, (**C**) total lipid, (**D**) low-density lipoprotein (LDL) cholesterol, (**E**) high-density lipoprotein (HDL) cholesterol, (**F**) free cholesterol, (**G**) free fatty acid (non-esterified fatty acid, NEFA), and (**H**) triglyceride (TG, neutral fat). Cont, HC, HC + G, HC + β, and HC + G + β indicate control, high-calorie-fed, high-calorie-fed + green-tea-intake, high-calorie-fed + β-cryptoxanthin-intake, and high-calorie-fed + green tea + β-cryptoxanthin-intake groups, respectively. Data are presented as mean ± standard deviation; * *p* < 0.05 compared to the control group, and ^#^ *p* < 0.05 compared to high-calorie-fed group.

Furthermore, the blood chemistry of mice in the control HC, HC + G, HC + β, and HC + G + β groups was examined. Mice in the HC, HC + G, and HC + β groups had significantly higher levels of blood glucose, free cholesterol, NEFA, and triglycerides compared to the control group (Figure 3A,F–H). However, the combined treatment with catechin and β-cryptoxanthin significantly restored these parameters to normal levels, with no significant difference between the control and HC + G + β groups (Figure 3A,F–H). Additionally, there was no significant difference in high-density lipoprotein (HDL) cholesterol levels between the control and treatment groups (Figure 3E). The HC group showed a significantly higher total serum protein level compared to the control group; however, the combined treatment with catechin and β-cryptoxanthin restored the obesity-induced increase in total protein level to normal, with no significant difference between the control and HC + G + β groups (Figure 3B). Total serum lipid level was significantly higher in the HC and HC + β groups compared to the control group; however, the combined treatment with catechin and β-cryptoxanthin restored the total lipid level to normal, with no significant difference between the control and HC + G + β groups (Figure 3C). There was a significant increase in LDL cholesterol levels in the HC group compared to the control group; however, treatment with catechin, β-cryptoxanthin, and catechin + β-cryptoxanthin restored LDL cholesterol levels to normal (Figure 3D).

### 3.3. Changes in Adipocyte Size and Adipocyte Inflammatory Response

**Figure 4:**
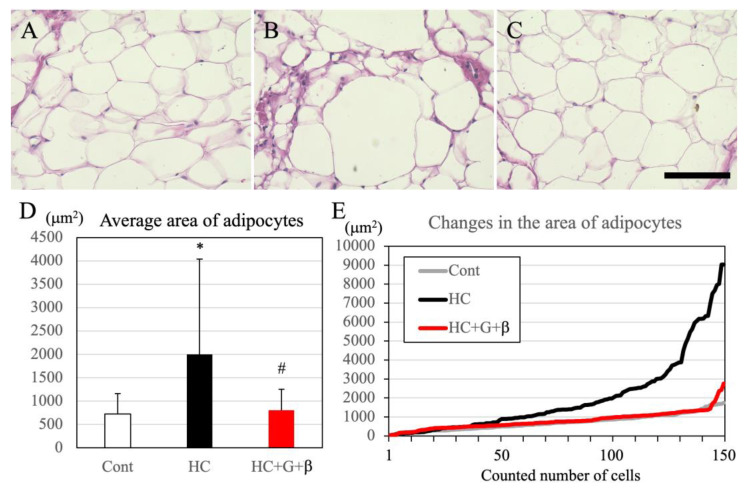
Hematoxylin–eosin (HE) staining of white adipocytes in the abdomen. (**A**) Adipocytes of mice in the control group, (**B**) adipocytes of mice in the high-calorie-fed group, (**C**) adipocytes of mice in the high-calorie-fed + green tea + β-cryptoxanthin-intake group. Scale bar is in C = 50 μm. (**D**) Average area of adipocytes. (**E**) Variation in the size of adipocytes. Cont, HC, and HC + G + β indicate the control, high-calorie-fed, and high-calorie-fed + green tea + β-cryptoxanthin-intake groups, respectively. Data are shown as mean ± standard deviation; * *p* < 0.05 compared to the control group, and ^#^ *p* < 0.05 compared to high-calorie-fed group.

The HE staining was performed to elucidate the effect of the treatments on adipocyte accumulation and inflammatory response. Compared to the control group, there was a significant increase in the size of white adipocytes in the HC group; however, combined treatment with catechin and β-cryptoxanthin restored adipocyte size to normal (Figure 4A–C). Additionally, we measured the size of a total of 150 adipocytes in each group to quantify adipocyte area. Adipocyte area was significantly higher in the HC group compared to the control group; however, combined treatment with catechin and β-cryptoxanthin significantly decreased the size of adipocytes compared with that in the HC group, with no significant difference in adipocyte area between the control and HC + G + β groups (Figure 4D). Changes in adipocyte area with an increasing number of adipocytes (50–150 adipocytes/group) are shown in Figure 4E. Consistent with the results of the HE staining, adipocyte area was significantly larger in the HC group relative to the control group; however, there was no significant difference in adipocyte area between the control and HC + G + β groups (Figure 4E).

**Figure 5:**
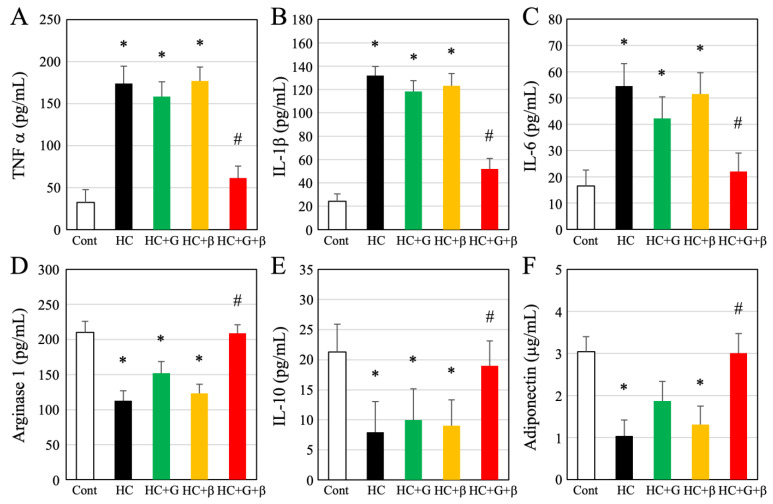
Quantitative analysis of inflammatory cytokines in adipose tissue using ELISA. (**A**) TNF-α, (**B**) IL-1β, (**C**) IL-6, (**D**) arginase 1 (ARG1), (**E**) IL-10, and (**F**) adiponectin. Cont, HC, HC + G, HC + β, and HC + G + β indicate the control, high-calorie-fed, high-calorie-fed + green-tea-intake, high-calorie-fed + β-cryptoxanthin-intake, and high-calorie-fed + green tea + β-cryptoxanthin-intake groups, respectively. Data are shown as mean ± standard deviation; * *p* < 0.05 compared to the control group, and ^#^ *p* < 0.05 compared to high-calorie-fed group.

The effects of the treatments on the inflammatory response in adipocytes were examined using ELISA. Compared to the control group, there was an increase in TNF-α, interleukin 1β (IL-1β), and IL-6 levels in the HC, HC + G, and HC + β groups (Figure 5A–C). In contrast, arginase 1 and IL-10 levels decreased significantly in the HC, HC + G, and HC + β groups compared to the control group (Figure 5D,E). However, combined treatment with green tea catechin and β-cryptoxanthin significantly restored TNF-α, IL-1β, IL-6, arginase 1, and IL-10 levels to normal, with no significant difference between the control and HC + G + β groups (Figure 5A–E). Additionally, adiponectin levels were significantly lower in the HC and HC + β groups compared to the control group (Figure 5F). However, treatment with catechin and combined treatment with catechin and β-cryptoxanthin significantly increased adiponectin levels compared to the HC and HC + β groups, and there was no significant difference between the control, HC + G, and HC + G + β groups (Figure 5F).

### 3.4. Reactions to Adipocytes in the Liver and Changes in Liver Structure and Function

**Figure 6:**
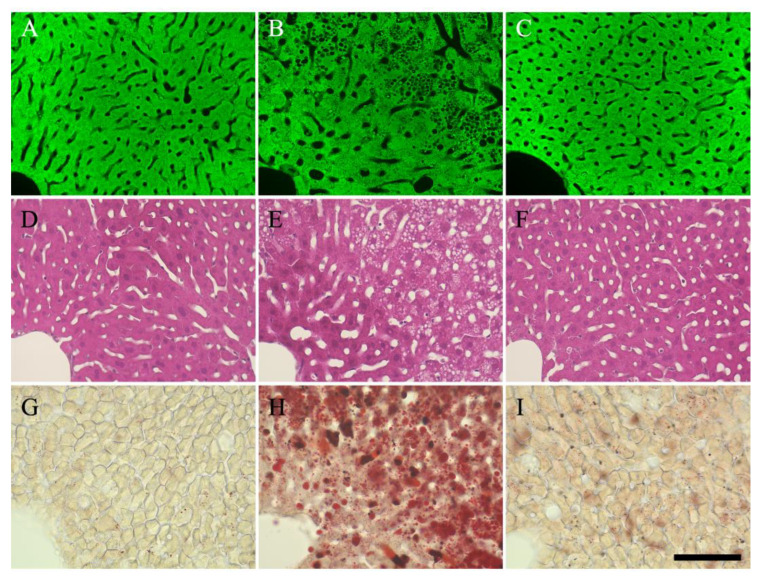
(**A**–**C**) Liver stained with adiponectin receptor, (**D**–**F**) liver stained with hematoxylin–eosin (HE), and (**G**–**I**) liver stained with Oil-Red-O. (**A**,**D**,**G**) Hepatocytes in the control group, (**B**,**E**,**H**) hepatocytes in the high-calorie-fed (HC) group, (**C**,**F**,**I**) hepatocytes in the high-calorie-fed + green tea + β-cryptoxanthin-intake (HC + G + β) group. Scale bar is in C = 100 μm.

To evaluate the hepatic reactions to adipokine, particularly adiponectin, we analyzed liver tissue samples from each group. The liver tissues were incubated with anti-adiponectin receptor antibody (Figure 6A–C), and adiponectin receptors were detected in hepatocytes in the control, HC, and HC + G + β groups (Figure 6A–C). Histopathological analysis was performed to detect morphological changes in hepatocytes using the HE staining. The liver of mice in the control group appeared healthy (Figure 6D). In contrast, abnormal morphological changes were observed in the tissues of mice in the HC group, with numerous vacuoles in the hepatocytes (Figure 6E). However, combined treatment with catechin and β-cryptoxanthin protected the liver against obesity-induced damage, with almost no vacuolar degeneration observed in the liver tissues of the mice (Figure 6F). Furthermore, Oil-Red O staining was performed to elucidate the effect of the treatment on lipid droplet accumulation (Figure 6G–I). Almost no lipid droplets were observed in hepatocytes in the control group (Figure 6G); in contrast, abnormal accumulation of lipid droplets was observed in the HC group (Figure 6H). However, combined treatment with catechins and β-cryptoxanthin ameliorated the abnormal accumulation of lipid droplets (Figure 6I).

**Figure 7:**
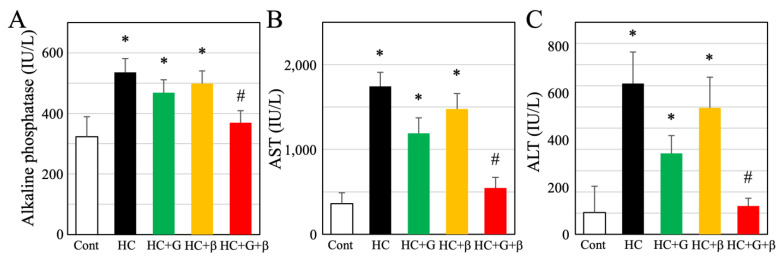
Serum levels of (**A**) alkaline phosphatase (ALP), (**B**) aspartate aminotransferase (AST), and (**C**) alanine aminotransferase (ALT). Cont, HC, HC + G, HC + β, and HC + G + β indicate the control, high-calorie-fed, high-calorie-fed + green-tea-intake, high-calorie-fed + β-cryptoxanthin-intake, and high-calorie-fed + green tea + β-cryptoxanthin-intake groups, respectively. Data are shown as mean ± standard deviation; * *p* < 0.05 compared to control animals, and ^#^ *p* < 0.05 compared to HC.

Serum ALP, AST, and ALT levels were examined to elucidate the effects of the treatments on hepatic function. Compared to the control group, serum ALP, AST, and ALT levels were significantly higher in the HC, HC + G, and HC + β groups (Figure 7A–C). However, combined treatment with catechins and β-cryptoxanthin significantly decreased ALP, AST, and ALT levels compared to the HC, HC + G, and HC + β groups, with no significant difference between the control and HC + G + β groups. Overall, these results showed that catechins and β-cryptoxanthin improved liver function.

**Figure 8:**
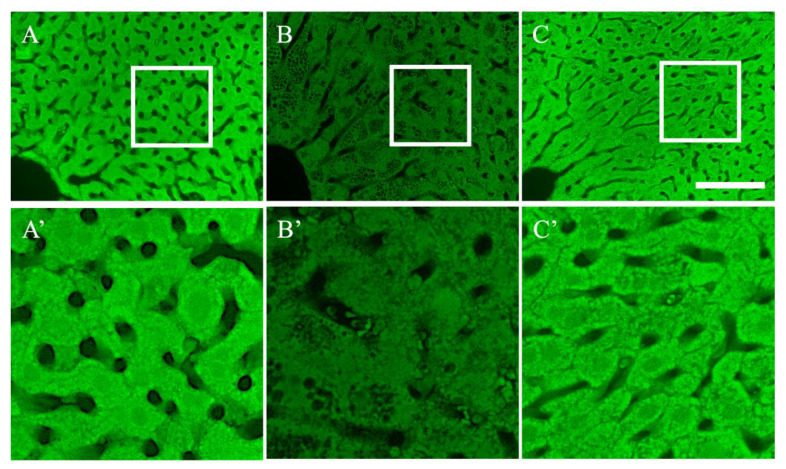
Photographs of liver stained with activin E ((**A**–**C**): low magnification views; (**A′**–**C′**): high magnification views). (**A**,**A′**) Hepatocytes of mice in the control group, (**B**,**B′**) hepatocytes of mice in the high-calorie-fed (HC) group, (**C**,**C′**) hepatocytes of mice in the high-calorie-fed + green tea + β-cryptoxanthin-intake (HC + G + β) group. Scale bar is in C = 100 μm.

Finally, we examined the expression of activin E, which is secreted from hepatocytes and promotes energy metabolism in adipose tissue (Figure 8). Activin E was uniformly expressed in the hepatocytes of mice in the control group (Figure 8A,A′). In contrast, although activin E was uniformly expressed in the liver of mice in the HC groups, the expression level was weak (Figure 8B,B′). In the liver of mice in the HC + G + β group, while activin E accumulation in the hepatocytes was sporadic (Figure 8C′), it was strongly expressed in almost all hepatocytes to the same extent as in the control mice (Figure 8C,C′).

## 4. Discussion

Recent findings suggest that tea catechins possess chronic obesity-suppressive effects [24,25]. However, it is impractical to apply the high concentrations or large quantities of tea catechins required to achieve such results in humans [24,25,36]. Additionally, long-term administration is necessary for the beneficial effects to be detected [37]. Therefore, there is a demand for foods that enhance the obesity-suppressing effects of tea catechins. A previous study showed that cryptoxanthin, which is present in citrus fruits, enhanced the obesity-suppressing effect of tea catechins in MSG model mice [29]. Although several studies have been performed on the pathogenesis and mechanism of obesity using the MSG model, chronic obesity in humans is mostly associated with high-calorie intake, making the MSG model unsuitable for extrapolating chronic obesity in humans. Therefore, it is necessary to verify whether the combined intake of tea catechin and β-cryptoxanthin can suppress high-calorie-intake-induced chronic obesity.

In the present study, we examined the antiobesity effect of catechins and β-cryptoxanthin in a mouse model of obesity caused by high-calorie intake. Compared to the control group, high-calorie intake caused a significant increase in the body weight of the mice, which was comparable to the body weight of mice in the MSG group (Figure 1B). A high-calorie diet is rich in fat and oil, which can induce fat accumulation and obesity. Serum biochemical analysis revealed that feeding a high-calorie diet affected serum parameters associated with obesity, including total glucose, NEFA, total cholesterol, and triglyceride. However, combined treatment with tea catechin and β-cryptoxanthin significantly improved the serum parameters (Figure 3), indicating that tea catechin and β-cryptoxanthin synergistically improved serum parameters in mice with obesity.

Tea catechin is a type of polyphenol that is found in large amounts in green tea and has been consumed in many countries for centuries. Consuming tea catechins with meals, particularly gallated catechins, has been demonstrated to reduce pancreatic lipase activity in the small intestine, which inhibits fat absorption and encourages fat excretion in the feces, leading to weight loss. Furthermore, gallated catechins bind to bile acid micelles in the small intestine and release the cholesterol from the micelles, thus decreasing cholesterol absorption and serum cholesterol levels [38]. It is believed that tea catechins are absorbed by the body and transported to target cells, where they act similarly to other bioactive substances [39]. Tea catechins can boost the expression and activity of lipolytic enzymes in fat cells, thus augmenting the release of fat-derived glycerol [40]. Furthermore, catechins have been demonstrated to boost the activity of hepatic β-oxidation-related enzymes, augment skeletal muscle β-oxidation-related enzymes and fatty acid transport enzymes, and increase β-oxidation activity when combined with exercise [28]. It is thought that increased fat burning and increased energy expenditure during everyday activities can help to balance energy and lipids and reduce visceral fat. Recent discoveries indicated that tea catechins can improve energy metabolism in brown adipocytes, potentially alleviating chronic obesity [40,41].

Of the eight types of catechins, the gallate-type catechins (epigallocatechin gallate, epicatechin gallate, gallocatechin gallate, and catechin gallate) are responsible for the effect of tea catechins on fat absorption, whereas the free-type catechins (epigallocatechin, epicatechin, gallocatechin, and catechin) are not effective. The gallate-type catechin is essential in inhibiting fat absorption [42,43]. It has been reported that long-term consumption of gallated catechins can significantly reduce body weight and visceral fat [44]. The tea catechin mix used in this study contains a sufficient amount of gallate-type catechins and is thought to have a positive impact on weight control (Figure 2). However, to achieve an antiobesity effect in humans, a daily intake of 200 mg or more is necessary, which is equivalent to around five cups of regular green tea [43]. At present, supplements and green tea with high catechin concentrations are available but not widely used.

Obesity is a chronic inflammatory disease associated with a decrease in the number of M2 macrophages and an increase in M1 macrophages in adipose tissue [45]. M1 macrophages secrete cytokines, such as TNF-α, that can cause adipocyte insulin resistance [46]. Overall, these results suggest that an increase in the proliferation of M1 macrophages is correlated with insulin resistance. A previous study has shown that both the number of M1 macrophages and inflammatory markers in adipose tissue decrease with weight loss [45]. Therefore, shifting the activation state of macrophages in adipose tissue from M1 to M2 could be a new therapeutic strategy for type 2 diabetes. The results of the present study showed that the combined intake of tea catechins and β-cryptoxanthin not only suppressed body weight gain but also reduced the number of M1 macrophages, suppressed the secretion of cytokines secreted from M1 macrophages, and promoted M2 macrophages (Figure 5). The results suggest that adipocyte function was improved by macrophage-mediated action. Until now, the molecular mechanism of chronic inflammation associated with obesity has mainly been analyzed by focusing on the activation mechanism of M1 macrophages that secrete inflammatory cytokines. It has been reported that obesity is caused by chronic inflammation due to the reduced activity of M2 macrophages, which have anti-inflammatory effects in adipose tissue [47]. However, whether M2 macrophage activities are attenuated in obesity, as well as the underlying molecular mechanism, remains unclear. This study shows that M2 macrophage activity is decreased in obesity, and that it is activated by the intake of tea catechins and β-cryptoxanthin. Recently, it was shown that the obesity-associated decreased expression of insulin receptor substrate-2 (Irs2) in macrophages attenuated the M2 macrophage activation by IL-4 [47]. Further studies are necessary to elucidate the specific mechanism of macrophages in tea catechin- and β-cryptoxanthin-induced improvements to facilitate the treatment of diabetes, chronic inflammatory diseases, cardiovascular diseases, and metabolic syndrome.

There was a significant increase in white adipose tissue, which can significantly increase adipokine secretion [14,48,49]. Adiponectin, which is one of the adipokines, has a variety of physiological activities that are beneficial to the body, such as restoring insulin to normal and preventing arteriosclerosis and cardiovascular diseases [50,51]. Moreover, it has been reported that AdipoR2, an adiponectin receptor, is strongly expressed in hepatocytes and promotes normal liver function, fatty acid decomposition, and energy metabolism [21,22,52]. In the present study, adiponectin expression in adipose tissues was significantly lower in mice with chronic obesity; however, combined treatment with tea catechins and β-cryptoxanthin restored normal adiponectin levels (Figure 5F). There was no significant difference in the expression and distribution of AdipoR2 between the groups (Figure 6A–C). However, these results suggest that hepatocytes are likely to be strongly influenced by adiponectin. Additionally, combined treatment with tea catechins and β-cryptoxanthin reversed obesity-induced increases in lipid droplet size and adipocyte area. Moreover, combined treatment with tea catechins and β-cryptoxanthin increased activin E biosynthesis (Figure 8), which is secreted from hepatocytes and induces energy metabolism. Although more research is needed, the findings of this study may be beneficial in developing medications aimed at enhancing energy metabolism to combat obesity.

It is anticipated that the bioregulatory properties of food will help to prevent and ameliorate diseases, including those associated with modern lifestyles that are on the rise. This approach is demonstrated by the ongoing development of functional food products globally. Furthermore, a prior study demonstrated that a combined treatment of green tea and citrus-derived polyphenols (over a period of 12 weeks) decreased body weight, enhanced BMI, and improved blood chemistry in humans [53]. In conclusion, these findings indicate that citrus-derived polyphenols, such as mandarin oranges, can enhance the therapeutic effects of green tea catechins. In our study, we utilized oranges containing β-cryptoxanthin, which is anticipated to have a more potent antioxidant effect than polyphenols derived from citrus fruits. The combination of green tea and tangerines containing β-cryptoxanthin, when taken for a brief period (4 weeks), had an antiobesity effect, with a lower intake of catechin than previously reported. It is noteworthy that the amount of β-cryptoxanthin and catechin needed to be taken in one day is quite small, so it is thought to be beneficial in combating obesity in humans.

In humans, obesity can be caused not only by excessive eating but also by dietary alterations, particularly the consumption of high-calorie foods. The outcomes of our prior study utilizing the MSG model [29] and the present study suggest the possibility of similar effects in humans with obesity. In conclusion, the results of this study can be used as a foundation for further research into the antiobesity mechanisms of tea catechins and clinical trials in humans.

## Data Availability

The datasets used and/or analyzed during this study are available from the corresponding author upon reasonable request.
